# Higher Leptin-to-Adiponectin Ratio Strengthens the Association Between Body Measurements and Occurrence of Type 2 Diabetes Mellitus

**DOI:** 10.3389/fpubh.2021.678681

**Published:** 2021-07-23

**Authors:** Pei-Ju Liao, Ming-Kuo Ting, I-Wen Wu, Shuo-Wei Chen, Ning-I Yang, Kuang-Hung Hsu

**Affiliations:** ^1^Master Degree Program in Healthcare Industry, Chang Gung University, Taoyuan, Taiwan; ^2^Division of Endocrinology and Metabolism, Chang Gung Memorial Hospital, Keelung, Taiwan; ^3^Division of Nephrology, Chang Gung Memorial Hospital, Keelung, Taiwan; ^4^Division of Gastroenterology and Hepatology, Chang Gung Memorial Hospital, Keelung, Taiwan; ^5^Division of Cardiology, Chang Gung Memorial Hospital, Keelung, Taiwan; ^6^Healthy Aging Research Center, Chang Gung University, Taoyuan, Taiwan; ^7^Laboratory for Epidemiology, Department of Healthcare Management, Chang Gung University, Taoyuan, Taiwan; ^8^Department of Emergency Medicine, Chang Gung Memorial Hospital, Taoyuan, Taiwan; ^9^Department of Urology, Chang Gung Memorial Hospital, Taoyuan, Taiwan; ^10^Research Center for Food and Cosmetic Safety, College of Human Ecology, Chang Gung University of Science and Technology, Taoyuan, Taiwan; ^11^Department of Safety, Health, and Environmental Engineering, Ming Chi University of Technology, Taipei, Taiwan

**Keywords:** limbs measurements, body measurements, adiponectin, leptin, type 2 diabetes mellitus

## Abstract

**Aim:** This case–control study aimed to investigate the interrelations of body measurements and selected biomarkers in type 2 diabetes mellitus (T2DM).

**Methods:** We recruited 98 patients with T2DM and 98 controls from 2016 to 2018 in Taiwan. Body measurements were obtained using a three-dimensional body surface scanning system. Four biomarkers related to insulin resistance, adipokines, and inflammation were assayed. A multiple logistic regression model was used to perform multivariable analyses.

**Results:** Four body measurements, namely waist circumference (odds ratio, OR = 1.073; 95% confidence interval, CI = 1.017–1.133), forearm circumference (OR = 1.227; 95% CI = 1.002–1.501), thigh circumference (OR = 0.841; 95% CI = 0.73–0.969), and calf circumference (OR = 1.25; 95% CI = 1.076–1.451), were significantly associated with T2DM. Leptin (OR = 1.09; 95% CI = 1.036–1.146) and adiponectin (OR = 0.982; 95% CI = 0.967–0.997) were significantly associated with T2DM. Six body measurement combinations, namely body mass index, waist-to-hip ratio, waist-to-height ratio, waist-to-thigh ratio, forearm-to-thigh ratio, and calf-to-thigh ratio (CTR), were significantly associated with T2DM. CTR had the strongest linear association with T2DM. Moderating effects of significant biomarkers, namely leptin and adiponectin, were observed. Participants with high leptin-to-adiponectin ratios and in the fourth CTR quartile were 162.2 times more prone to develop T2DM.

**Conclusions:** We concluded that a combination of leptin and adiponectin modulated the strength of the association between body measurements and T2DM while providing clues for high-risk group identification and mechanistic conjectures of preventing T2DM.

## Introduction

Type 2 diabetes mellitus (T2DM) is a rapidly burgeoning chronic disease that causes complications resulting in increased healthcare burden and affecting patient quality of life (1, 2). Studies have demonstrated that central obesity, or abdominal visceral fat accumulation, predominantly indicates T2DM risk and is associated with inflammatory response mechanisms and insulin resistance (3). A meta-analysis indicated that body mass index (BMI), waist circumference (WC), and waist-to-hip ratio (WHR) are three major body shape markers associated with T2DM incidence (4). Another meta-analysis indicated that waist-to-height ratio (WHtR) was superior to BMI in predicting diabetes and several other cardiometabolic risk factors (5). Other studies have revealed that BMI, WC, WHR, and WHtR may predict diabetes occurrence. WC is an indicator of abdominal visceral fat accumulation and is associated with insulin resistance and cardiometabolic risk (6–9). In addition, researchers have reported that thigh circumference (TC) or waist-to-thigh ratio (WTR) was associated with T2DM (10–12). In particular, a small TC, such as low subcutaneous fat or low skeletal muscle in the thigh has been recognized as a risk factor for hyperlipidemia and hyperglycemia (13). Body measurements provide information related to adverse or protective effects of T2DM. Although studies have indicated associations between T2DM and selected body measurements, such as WC, BMI, and body weight, comprehensive whole body measurements have seldom been addressed. Therefore, the association among body measurements, T2DM, and biomarkers related to insulin resistance and inflammation requires clarification.

Several biomarkers related to insulin resistance and inflammation demonstrated correlations with T2DM. Most documented biomarkers were associated with adipocytokines secreted by adipocytes and macrophages and migrated to the adipose tissue. Leptin involves the regulation of satiety and body weight and is positively associated with obesity, fat mass, insulin resistance, triglyceride levels, and inflammatory cytokines (5, 14). Although an association between leptin and T2DM was reported in Caucasian populations (7, 8), its effect was less obvious when insulin resistance and other confounding variables were included into the analysis (8). Adiponectin is negatively associated with obesity and involved in lipid clearance (5, 14). A meta-analysis reported a relative risk of 0.72 in developing T2DM per 1-log mg/ml increments in adiponectin level (6). High serum levels of pro-inflammatory biomarkers, such as tumor necrosis factor-alpha, interleukin-6, and C-reactive protein (hsCRP), were associated with a high risk of T2DM. Insulin-like growth factor (IGF) shares a structural homology with insulin, and increased blood levels of IGF-I were observed to be associated with T2DM in epidemiological studies (15, 16).

Earlier studies have taken body measurements using non-invasive three-dimensional (3D) scanning technology and demonstrated their association with T2DM (11, 17, 18). However, associations between body measurements and selected biomarkers of T2DM have not been explored thoroughly to date. Based on previous studies (11, 13, 17, 19–21), limb measurements, in addition to WC, may represent a feature for one's risk to T2DM. We hypothesize that selected biomarkers may strengthen the effect of a combination of body measurements, such as trunk and limb measurements, on T2DM. Therefore, this case–control study investigated the inter-relations between selected body measurements and recognized biomarkers on T2DM risk.

## Materials and Methods

### Study Samples

In total, 196 participants (98 with T2DM and 98 non-DM controls) were recruited from the Department of Health Promotion and Examination of Chang Gung Memorial Hospital in Northern Taiwan, representing a normal Taiwanese population. A 1:1 matching was performed with the same sex and age (±5 years) for each case–control pair from a 3D cohort visiting the hospital from March 2016 to January 2018. A minimal sample size of 90 for each group was calculated based on the 1:1 case–control design (α = 0.05, 1–β = 0.8, and odds ratio, OR = 2.5) according to previous publications (17, 22). Cases were ascertained by physicians of the endocrinology and metabolism department in the community hospital (Chang Gung Memorial Hospital). All T2DM cases included in this study were receiving treatment for blood sugar control for at least a year. The medication used for the treatment of T2DM included biguanides, alpha-glucosidase inhibitors, sulfonylureas, meglitinides, TZDs, DPP-4 inhibitors, SGLT-2 inhibitors, GLP-1 agonists, and insulin. Patients with comorbidities or complications, such as hypertension, cardiovascular disease, renal disease (chronic kidney disease, CKD, stages III, IV, V, and end-stage renal disease, ESRD), liver cirrhosis, chronic hepatitis, cancer, stroke, and disabilities, were excluded. The health status of controls was ascertained using questionnaires; data on medication and disease history and health check-up, such as fasting blood sugar (AC sugar), post-prandial sugar (PC sugar), and hemoglobin A1c (HbA_1_C) levels, were collected. Those who were not taking medications and without disease were included as healthy controls in this study. T2DM diagnosis was based on the following American Diabetes Association (ADA) guidelines: fasting for ≥8 h, AC sugar level ≥126 mg/dl, HbA_1_C level ≥6.5%, and PC sugar level (≥2 h) ≥200 mg/dl for two consecutive examinations. As confirmed by their medical records, those with no major illness or complications, namely, hypertension, cardiovascular disease, heart disease, renal disease, liver cirrhosis, or chronic hepatitis, were recruited as participants. This study was approved by the Institutional Review Board of Chang Gung Medical Foundation (107-0011C).

### Anthropometric Parameters

3D body surface measurements were obtained through whole body 3D laser scanning according to previously published methods (17, 19). The 3D laser scanning machine (LT3DCam) was designed by Logistic Technology Company (LTC, Hsinchu, Taiwan) and was proved to have a high standard of accuracy because of objective and comprehensive methods of measuring the human body surface. The standard procedure of measurement requires a participant to remove all outer clothes except for underclothing in preparation for scanning (women with bras in addition to pants). The participants were to stand still on the stage for scanning (total scanning time is ~10 s). In addition to body height and body weight, 22 one-dimensional measurements from four anatomical regions were obtained. The definition of each body measurement was adapted from previous research studies (17, 19) (Supplementary Material). The head and neck region included circumferences of the head and neck. The trunk region included chest circumference, chest width, WC, and waist width. The area from the hip to lower limbs included hip circumference, hip width, left leg length, right leg length, left TC, right TC, left calf circumference, and right calf circumference. The upper limb region included left arm length, right arm length, left upper arm circumference, right upper arm circumference, left forearm circumference, and right forearm circumference. In addition to frequently documented T2DM-related measurement combinations, such as BMI, WHR, WHtR, and WTR, we performed a backward selection from the 22 one-dimensional measurements, adjusting for age, sex, occupation, education, marriage, smoking, alcohol drinking, tea drinking, coffee drinking, betel nut chewing, and daily activity level. Combinations, such as forearm circumference to thigh circumference ratio (forearm to thigh ratio, FATR) and calf circumference to thigh circumference ratio (calf to thigh ratio, CTR), derived from significant body measurements by performing multivariable regression analysis, were used to evaluate their effects on T2DM and modulating effects of the selected biomarkers.

### Assays for Biomarkers

An enzyme-linked immunosorbent assay (ELISA) was used to quantify the concentration of serum biomarkers. The serum concentrations of leptin and adiponectin were assayed using commercial ELISA kits from Boster (Pleasanton, CA, United States). IGF-1 was determined using commercial ELISA kits from BioOcean (Shoreview, MN, United States). The hsCRP level was assayed using a commercial ELISA kit from Roche (Basel, Switzerland).

### Data Collection

A questionnaire was used to collect the following information: date of birth; sex; education; marital status; occupation; history of cigarette smoking, alcohol drinking, betel nut chewing, tea drinking, and coffee drinking; personal history of the disease (namely, diabetes, hypertension, heart disease, CKD, liver cirrhosis, and chronic hepatitis); and family history of T2DM. For those with no history of diabetes, a fasting blood glucose level was obtained. Diabetes was defined according to the ADA guidelines. For those with no history of hypertension, blood pressure was measured using a mercury sphygmomanometer on the left arm after a patient had rested for 20 min in a seated position. Hypertension was defined according to the guidelines of the Joint National Committee on Detection, Evaluation, and Treatment of High Blood Pressure (systolic blood pressure ≥ 140 mm Hg, diastolic blood pressure ≥ 90 mm Hg, or the use of antihypertensive medication) (23).

### Statistical Analyses

Two independent sample *t-*tests were performed to compare differences between continuous variables of the groups, and results are presented as the mean ± SD. The χ^2^-test was performed to differentiate between the distribution of categorical variables, and the results are expressed as frequencies and percentages between groups. 3D body surface measurements were screened by a two-sample *t-*test by comparing differences between the patients and controls. To avoid collinearity in the regression analysis, one body measurement with the lowest *p*-value was selected from each anatomic dimension for subsequent multivariable analysis. A logistic regression model was used to determine the strength of the association between the selected body measurements and T2DM. In addition to the forced-in sociodemographic and lifestyle variables, a backward model selection with *p* <0.2 was used to determine variables, namely, body measurements and biomarkers, to be retained in the regression model. The modulating effect was examined by comparing models with and without biomarkers while calculating the strength of association (OR) between the body measurement combinations and the T2DM. SPSS 22.0 statistical software (IBM Corporation, Armonk, NY, United States) was used for performing the analyses in this study.

## Results

Patients with type 2 diabetes mellitus (T2DM) had a lower level of education than those in the non-DM controls. T2DM was associated with occupational categories in which farmers and laborers, self-employed workers, and service industry workers were observed to have a high risk. Among the lifestyle variables, cigarette smoking, betel nut chewing, and low to medium activity levels were associated with the high risk of T2DM (Table 1).

**Table 1:** Sociodemographic and lifestyle variables of the study participants.

	**Control participants (*n* = 98)**	**DM patients (*n* = 98)**	***p*-value**
**Demographics**			
Age	58.68 ± 11.01	56.40 ± 10.60	0.141
Gender			1.000
Female	41 (41.8%)	41 (41.8%)	
Male	57 (58.2%)	57 (58.2%)	
Education			<0.0001
Elementary and below	21 (21.4%)	38 (38.8%)	
Junior high school	9 (9.2%)	20 (20.4%)	
Senior high school	29 (29.6%)	28 (28.6%)	
College/university and above	39 (39.8%)	12 (12.2%)	
Marital status			0.830
Married or coupled	85 (86.7%)	86 (87.8%)	
Unmarried	13 (13.3%)	12 (12.2%)	
Occupation			<0.0001
Government	13 (13.3%)	5 (5.1%)	
Farmers and laborers	12 (12.2%)	20 (20.4%)	
Business	19 (19.4%)	2 (2.0%)	
Self-employment	17 (17.3%)	26 (26.5%)	
Service industry	12 (12.2%)	24 (24.5%)	
Others	25 (25.5%)	21 (21.4%)	
**Lifestyle variables**			
Cigarette smoking			0.046
No	73 (74.5%)	60 (61.2%)	
Yes	25 (25.5%)	38 (38.8%)	
Alcohol drinking			0.133
No	69 (70.4%)	59 (60.2%)	
Yes	29 (29.6%)	39 (39.8%)	
Tea drinking			0.381
No	42 (42.9%)	36 (36.7%)	
Yes	56 (57.1%)	62 (63.3%)	
Coffee drinking			0.368
No	31 (31.6%)	37 (37.8%)	
Yes	67 (68.4%)	61 (62.2%)	
Betel nut chewing			0.013
No	95 (96.9%)	86 (87.8%)	
Yes	3 (3.1%)	12 (12.2%)	
Activity level			0.023
Low	50 (51.0%)	48 (49.0%)	
Median	31 (31.6%)	44 (44.9%)	
High	17 (17.3%)	6 (6.1%)	

The results of most of the selected body measurements were statistically significant between the cases and controls. In general, the patients with T2DM had larger body measurements than those in the controls, except for body height, head circumference, hip width, arm length, and leg length. The results of the multivariable analysis indicated that WC (OR = 1.073; 95% CI = 1.017–1.133), left forearm circumference (OR = 1.227; 95% CI = 1.002–1.501), right TC (OR = 0.841; 95% CI = 0.73–0.969), and right calf circumference (OR = 1.25; 95% CI = 1.076–1.451) were significantly associated with T2DM after adjusting for age, sex, education, marital status, occupation, smoking, alcohol drinking, coffee drinking, betel nut chewing, and daily activity level. The following six selected combinations were significantly associated with T2DM in the multivariable logistic regression analysis: BMI (OR = 1.318; 95% CI = 1.171–1.483), WHR (OR = 1.109; 95% CI = 1.046–1.176), WHtR (OR = 1.162; 95% CI = 1.086–1.243), WTR (OR = 1.534; 95% CI = 1.229–1.913), FATR (OR = 1.12; 95% CI = 1.047–1.197), and CTR (OR = 1.142; 95% CI = 1.075–1.214) (Table 2).

**Table 2:** Comparison of body measurements between the study groups.

**Stage 1: body measurements**	**Control participants (*n* = 98)**	**DM patients (*n* = 98)**	***p*-value**	OR (95% CI)^*^
**Whole body**				
Height (cm)	163.1 ± 9	161.7 ± 8.6	0.249	
Weight (kg)	64.6 ± 13.1	72.6 ± 13.1	<0.0001	
**Head and neck**				
Head circumference (cm)	56.9 ± 2.9	57.3 ± 2.7	0.292	
Neck circumference (cm)	40.6 ± 4.3	42.8 ± 3.8	<0.0001	
**Trunk**				
Chest width (cm)	31.8 ± 3.3	33.6 ± 2.8	<0.0001	
Chest circumference (cm)	95.6 ± 9.6	102.9 ± 9.9	<0.0001	
Waist width (cm)	31 ± 3.2	33.1 ± 3.1	<0.0001	
Waist circumference (cm)	86.0 ± 11.4	95.6 ± 11.6	<0.0001	1.073 (1.017, 1.133)
**Hip**				
Hip width (cm)	34.6 ± 2.6	34.9 ± 2.4	0.510	
Hip circumference (cm)	94.4 ± 8.4	100.1 ± 9.1	<0.0001	
**Upper limbs**				
**Arm length (cm)**				
Left	52.9 ± 3.7	53.0 ± 4.1	0.893	
Right	53.1 ± 3.6	53.2 ± 4.0	0.878	
**Upper arm circumference (cm)**				
Left	30.5 ± 2.9	31.8 ± 3.3	0.002	
Right	30.5 ± 2.9	32.0 ± 3.2	0.001	
**Forearm circumference (cm)**				
Left	21.7 ± 2.9	24.2 ± 3.2	<0.0001	1.227 (1.002, 1.501)
Right	22.1 ± 3.0	24.5 ± 3.3	<0.0001	
**Lower limbs**				
**Leg length (cm)**				
Left	69.1 ± 5.1	68.0 ± 4.4	0.095	
Right	69.1 ± 5.0	68.0 ± 4.3	0.079	
**Thigh circumference (cm)**				
Left	50.6 ± 4.2	52.1 ± 5.1	0.020	
Right	50.6 ± 4.1	52.2 ± 5.1	0.013	0.841 (0.730, 0.969)
**Knee circumference (cm)**				
Left	38.5 ± 2.8	39.8 ± 3.7	0.005	
Right	38.6 ± 2.7	39.9 ± 3.7	0.006	
**Calf circumference (cm)**				
Left	30.7 ± 4.1	34.4 ± 4.4	<0.0001	1.250 (1.076, 1.451)
Right	30.8 ± 4.1	34.6 ± 4.5	<0.0001	
**Stage 2: measurement combinations**				
BMI	24.1 ± 3.3	27.8 ± 4.5	<0.0001	1.318 (1.171, 1.483)
WHR ×100	91.1 ± 8.0	95.3 ± 5.6	<0.0001	1.109 (1.046, 1.176)
WHtR ×100	52.7 ± 5.9	59.3 ± 8.0	<0.0001	1.162 (1.086, 1.243)
WTR ×10	17.0 ± 2.1	18.4 ± 1.8	<0.0001	1.534 (1.229, 1.913)
FATR ×100	43.4 ± 6.2	46.6 ± 6.7	0.001	1.120 (1.047, 1.197)
CTR ×100	61.2 ± 8.0	66.3 ± 6.3	<0.0001	1.142 (1.075, 1.214)

*BMI, body mass index; WHR, waist-to-hip ratio; WHtR, waist-to-height ratio; WTR, waist-to-thigh ratio; FATR, forearm-to-thigh ratio; CTR, calf-to-thigh ratio*.

* *ORs obtained from a multivariate model included age, sex, occupation, education, marriage, smoking, alcohol drinking, tea drinking, coffee drinking, betel nut chewing, daily activity level, and investigated variables (stage 1: included waist circumference, left forearm circumference, right thigh circumference, and right calf circumference; stage 2: only one combination was included in each model)*.

The association between the four selected biomarkers and T2DM was examined, and the association between two biomarkers, namely, leptin and adiponectin, and T2DM was statistically significant in the univariate analysis. Leptin and adiponectin were observed to be significantly associated with T2DM in backward selection modeling, with ORs of 1.09 (95% CI = 1.036–1.146) and 0.982 (95% CI = 0.967–0.997), respectively (Table 3).

**Table 3:** The distribution of biomarkers between the study groups.

	**Control participants (*n* = 98)**	**DM patients (*n* = 98)**	***p*-value**	OR (95% CI)^*^	OR (95% CI)^**^
Leptin (ng/ml)	7.13 ± 7.26	12.88 ± 12.86	<0.0001	1.096(1.042,1.154)	1.090 (1.036, 1.146)
Adiponectin (μg/ml)	34.75 ± 36.25	22.13 ± 23.23	0.004	0.981 (0.968, 0.995)	0.982 (0.967, 0.997)
HSCRP (mg/l)	1.36 ± 1.78	1.79 ± 2.47	0.309	1.130 (0.946, 1.351)	
IGF (pg/ml)	28.39 ± 30.22	34.00 ± 45.36	0.162	1.001 (0.992, 1.011)	

* *Model includes age, sex, occupation, education, marriage, smoking, alcohol drinking, tea drinking, coffee drinking, betel nut chewing, daily activity level, and investigated variable (only one biomarker was included in each model)*.

** *Biomarkers were selected based on p < 0.2 with backward model selection in which the model includes age, sex, occupation, education, marriage, smoking, alcohol drinking, tea drinking, coffee drinking, betel nut chewing, daily activity level, leptin, and adiponectin*.

**Figure 1:**
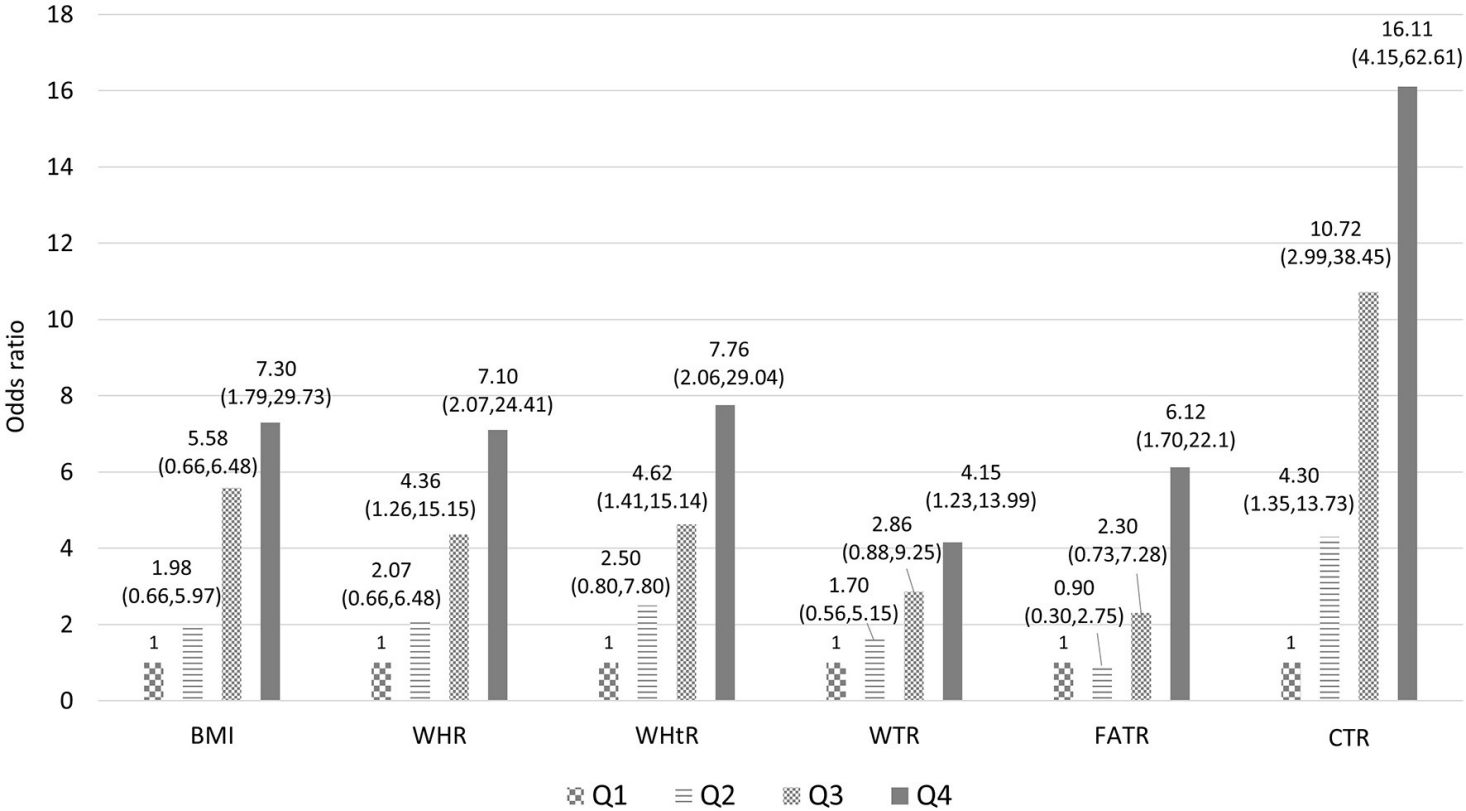
Association between the selected combination of body measurements and T2DM (model includes age, sex, occupation, education, marriage, smoking, alcohol drinking, tea drinking, coffee drinking, betel nut chewing, daily activity level, adiponectin, leptin, and quartile of selected body measurements combination. BMI body mass index; WHR, waist-to-hip ratio; WHtR, waist-to-height ratio; WTR, waist-to-thigh ratio; FATR, forearm-to-thigh ratio; CTR, calf-to-thigh ratio).

Six body measurement combinations were tested accordingly and then further categorized into quartiles to determine the monotonic trend association with T2DM. The results demonstrated a monotonic trend in multivariable-adjusted ORs for BMI (Q1 = 1, Q2 = 1.98, Q3 = 5.58, and Q4 = 7.3), WHR (Q1 = 1, Q2 = 2.07, Q3 = 4.36, and Q4 = 7.1), WHtR (Q1 = 1, Q2 = 2.5, Q3 = 4.62, and Q4 = 7.76), WTR (Q1 = 1, Q2 = 1.17, Q3 = 2.86, and Q4 = 4.15), FATR (Q1 = 1, Q2 = 0.9, Q3 = 2.3, and Q4 = 6.12), and CTR (Q1 = 1, Q2 = 4.3, Q3 = 10.72, and Q4 = 16.11). The highest strength of association was found between CTR and T2DM, followed by WHtR, BMI, and WHR (Figure 1). When leptin, adiponectin, and leptin-to-adiponectin ratios were stratified into median, high, and low, different moderating effects were found. The subgroup with higher leptin levels had multivariable-adjusted ORs of 1.03 (95% CI = 0.16–6.53), 3.94 (95% CI = 0.6–26), 19.86 (95% CI = 3.09–127.69), and 30.39 (95% CI = 4.43–208.71) with CTRs at Q1, Q2, Q3, and Q4, respectively, when compared with the subgroup with lower leptin and in the first CTR quartile (Q1) (Model 1, Table 4). The subgroup with lower adiponectin levels had multivariable-adjusted ORs of 3.09 (95% CI = 0.46–20.73), 8.82 (95% CI = 1.46–53.15), 27.74 (95% CI = 4.38–175.82), and 32.25 (95% CI = 4.37–237.9) with CTRs at Q1, Q2, Q3, and Q4, respectively, when compared with the subgroup with higher adiponectin and in the first CTR quartile (Q1) (Model 2, Table 4). The subgroup with higher leptin-to-adiponectin ratios had multivariable-adjusted ORs of 12.6 (95% CI = 1.78–89.27), 45.79 (95% CI = 6.28–333.69), 55.51 (95% CI = 7.83–393.53), and 162.2 (95% CI = 17.17–1534.37) with CTRs at Q1, Q2, Q3, and Q4, respectively, when compared with the subgroup with a lower leptin-to-adiponectin ratio and in the first CTR quartile (Q1) (Model 3, Table 4).

**Table 4:** Stratified analysis between CTR quartile and leptin, adiponectin, and leptin-to-adiponectin ratio (high/low) on T2DM.

	Model 1^*^						Model 2^*^						Model 3^*^					
	**Leptin**						**Adiponectin**						**Leptin-to-adiponectin ratio**					
**CTR Quartile**	**Low**			**High**			**High**			**Low**			**Low**			**High**		
	***n***	**OR**	**95% CI**	***n***	**OR**	**95% CI**	***n***	**OR**	**95% CI**	***n***	**OR**	**95% CI**	**n**	**OR**	**95% CI**	**n**	**OR**	**95% CI**
Q1	14	1.00	-	34	1.03	(0.16, 6.53)	29	1.00	-	19	3.09	(0.461, 20.73)	27	1.00	-	21	12.60	(1.78, 89.27)
Q2	31	3.56	(0.67, 18.95)	19	3.94	(0.60, 25.99)	23	8.54	(1.45, 50.19)	27	8.82	(1.46, 53.15)	24	6.22	(0.94, 41.05)	26	45.79	(6.28, 333.69)
Q3	24	7.20	(1.18, 43.99)	25	19.86	(3.09, 127.69)	21	11.99	(1.79, 80.49)	28	27.74	(4.38, 175.82)	21	66.37	(7.96, 553.75)	28	55.51	(7.83, 393.53)
Q4	28	8.52	(1.29, 56.49)	21	30.39	(4.43, 208.71)	24	26.80	(4.17, 172.27)	25	32.25	(1.02, 1.15)	25	39.85	(5.49, 289.27)	24	162.20	(17.15, 1534.37)

*CTR, calf-to-thigh ratio*.

* *All the models are adjusted for age, sex, occupation, education, marital status, smoking, alcohol drinking, tea drinking, coffee drinking, betel nut chewing, daily activity level, leptin, and adiponectin excluding the investigated variable per se. Interactive effects between CTR and the biomarkers (leptin, adiponectin, and leptin-to-adiponectin ratio) were proved with significance level p < 0.001*.

## Discussion

The aim of this study was to investigate the interrelations of body measurements and selected biomarkers with T2DM. The results of this case–control study demonstrated that WC, left forearm circumference, right TC, and right calf circumference were associated with T2DM, whereas the biomarkers leptin and adiponectin were correlated with T2DM. In addition, the results indicated that CTR exhibited the highest strength of positive linear association with T2DM among the six selected body measurement combinations and was modulated by leptin, adiponectin, and the ratio of leptin to adiponectin in the multiple regression analysis. Although the literature indicated that TC is negatively associated with the incidence and prevalence of T2DM (20), this study indicated that calf circumference was another marker associated with T2DM and can interact with leptin and adiponectin mechanistically. Based on the findings of this study, a combination of WC or limb circumference measurements and leptin/adiponectin may be used to represent the risk of T2DM while providing evidence for intervention strategies to prevent the disease among high-risk groups. In future clinical or epidemiological practices, people with high values of the six selected body measurement combinations may be considered as high-risk groups for the occurrence and adverse progression of T2DM. Intensive interventions, such as exercise and nutrition, or examinations, such as leptin and adiponectin, are suggested for high-risk individuals.

The associations between waist or limb measurement combinations and T2DM may be attributed to the secretion by adipocytes and macrophages that have migrated to the adipose tissue, which activates adipocytokines such as adiponectin and leptin (3–5, 24). Among them, leptin is positively associated with obesity, fat mass, insulin resistance, triglyceride levels, and inflammatory cytokines and negatively associated with high-density lipoprotein cholesterol (5). Although a positive association of leptin with T2DM has been demonstrated in Caucasian populations (7, 8), its effect was unknown when body measurements and other confounders were taken into account. An earlier study demonstrated that high leptin levels were associated with a low risk of diabetes after adjusting for obesity, adiponectin, triglyceride, hypertension, and inflammation scores (8). Previous research demonstrated the association between central obesity and T2DM. However, in this study, limb measurements played an important role in the disease status, wherein a synergistic effect of adipokines on T2DM was observed. The thigh muscular tissue was observed to excrete proteins against insulin resistance and inflammation. An adverse combination of limb measurements may imply a lack of protective effects, especially in a state of leptin resistance, which is associated with high chance of insulin resistance and T2DM (25). The results of this study showed a synergistically interactive effect of leptin levels and body measurements, such as central obesity and limbs measurements, on T2DM, providing an in-depth observation of future mechanistic and preventive methods.

The findings on the association between limb measurements and T2DM are the most notable, whereas levels of leptin and adipokines played a moderating role. A large TC is generally regarded as a protective factor for T2DM in both cross-sectional and longitudinal studies (20). As observed in the literature, thigh skeletal muscles are the key target organs for insulin action and sites of insulin resistance (21). A low muscle mass or less subcutaneous fat in the thighs is believed to be associated with hyperglycemia and diabetes by the action of insulin resistance (13). The association between the forearm/thigh or calf/thigh combination and T2DM is partly explained by a small TC, and the mechanism of a large forearm or calf circumference in T2DM warrants further discussion.

Whether changes in plasma adipokines and/or inflammatory parameters observed in patients with T2DM are because of excessive adipose tissue and/or direct association with diabetes status is as yet unclear (21, 26). Earlier studies have demonstrated that circulating leptin levels were high in obese individuals and in patients with metabolic syndrome (27). Earlier reports have demonstrated that elevated leptin concentrations in obese participants were directly proportional to obesity and positively correlated with body fat mass. In contrast, hyperinsulinemia may, in turn, exacerbate obesity and further increase leptin levels, resulting in a positive feedback loop that promotes the development of diabetes (25, 28). Therefore, the results demonstrated a close relationship between T2DM and leptin levels and with adverse body measurements.

Leptin is assumed to be elevated by unfavorable body measurements, which indicate that fatty cells accumulate and, thus, reduce insulin sensitivity, possibly resulting in decreased glucose tolerance (29). These observations suggest that the independent role of high leptin levels in predicting the risk of diabetes can be because of the role of leptin in regulating insulin sensitivity and secretion. Moreover, adverse body measurements exacerbate the insulin resistance loop (30).

This study demonstrated an inverse relationship between adiponectin and T2DM in the multivariate regression analysis. The relationship between adiponectin and insulin sensitivity varies among ethnicities. In a multiethnic population-based study, adiponectin levels were negatively correlated with insulin resistance only in the Caucasian population, whereas no correlation was observed in Black and South Asian populations (31). This study demonstrated that the association between adiponectin and insulin sensitivity may be because of body shape differences. Adiponectin differs from other adipokines in that it is inversely proportional to obesity and even the distribution of body adipocytes (32, 33). We hypothesized that the discrepancy depends on the body shape distribution in the investigated population. Furthermore, the observations of the authors suggested that the role of adiponectin in T2DM can involve other biomarkers, such as leptin, as well as body measurements, such as limb circumference, in this Asian population. As per the results of this study, the ratio of leptin to adiponectin should be considered an important marker to estimate adverse body measurements of an individual as a risk for T2DM.

This study used a case–control design to explore the interrelations among body measurements, biomarkers, and T2DM. While relationships between T2DM and individual body measurements or biomarkers have been broadly explored, the interactive effect between body measurements and biomarkers in T2DM was rarely verified. Most notably, this study discloses the highest likelihood of T2DM among the participants with both higher CTR and leptin-to-adiponectin ratio, which provides a roomy discussion for the mechanistic pathway of T2DM in the future. To increase the accuracy and comprehensiveness of body measurements, we used accurate measuring techniques such as 3D whole-body scanning, computer-based technology (which reduced measurement errors), biomarker analysis (which anchored biopathways and mechanisms), and multivariable model construction (which was more comprehensive). Nevertheless, this study has certain limitations. First are the limitations of a case–control design as opposed to a cohort study generally applied to this study, particularly on temporal ambiguity. Second, measurements of 3D whole body scanning were obtained only once; therefore, we did not count body measurement changes over time. Third, we selected one side with a lower *p*-value among the limb measurements for further combination analyses. We did not exercise the effects of choosing an alternative side for the combinations. Fourth, the study population was of Asian ethnicity; therefore, the findings may apply only to people in Asia (such as in China). Caution should be taken when generalizing the results to Western populations. Fifth, the DM vintage and treatment history were not included in the analysis, which may be confounded by disease progression or severity. Finally, this was a hospital-based sampling design that may need confirmation with community-based samples.

## Conclusions

In addition to body measurements, such as WC, TC, forearm circumference, and calf circumference, this study demonstrated leptin and adiponectin, and their combinations to be associated with T2DM. The CTR exhibited the strongest association with T2DM, whereas the ratio of leptin to adiponectin heightened the strength of the association with T2DM. The body measurements and significant biomarkers obtained in this study can provide mechanistic conjectures for high-risk group identification and prevention of T2DM in future practice.

## Data Availability Statement

The datasets presented in this article are not readily available because Regulation of Ministry of Health and Welfare in Taiwan. Requests to access the datasets should be directed to Kuang-Hung Hsu (khsu@mail.cgu.edu.tw).

## Ethics Statement

The studies involving human participants were reviewed and approved by Institutional Review Board of Chang Gung Medical Foundation. The patients/participants provided their written informed consent to participate in this study.

## Author Contributions

P-JL and K-HH: study concept, design, statistical analysis, and drafting. M-KT, I-WW, S-WC, N-IY, and K-HH: acquisition of data, funding obtainment, administrative, technical, or material support. P-JL, M-KT, and K-HH: analysis and interpretation of data. K-HH: supervision. All authors contributed to the article and approved the submitted version.

## Conflict of Interest

The authors declare that the research was conducted in the absence of any commercial or financial relationships that could be construed as a potential conflict of interest.

## Publisher's Note

All claims expressed in this article are solely those of the authors and do not necessarily represent those of their affiliated organizations, or those of the publisher, the editors and the reviewers. Any product that may be evaluated in this article, or claim that may be made by its manufacturer, is not guaranteed or endorsed by the publisher.
