# Supplementary material for: Higher Leptin-to-Adiponectin Ratio Strengthens the Association Between Body Measurements and Occurrence of Type 2 Diabetes Mellitus
Source: Front Public Health. 2021 Jul 23;9:678681. doi: 10.3389/fpubh.2021.678681 (PMC8342761; doi:10.3389/fpubh.2021.678681)
Supplement: Supplementary file 1 [file Table_1.docx]

Supplementary 1. The definition of the size calculation of 3D human body data*

| Body measurements | Definition |
| --- | --- |
| **Head and neck** |  |
| Head circumference (cm) | The length of the horizontal section profile on the most protrusion of forehead, which is processed as a convex hull. |
| Neck circumference (cm) | The length of the horizontal section profile on the midway of the neck between mid-cervical spine and mid-anterior neck, just below the laryngeal prominence, which is processed as a convex hull. |
| **Trunk** |  |
| Chest width (cm) | The width of the horizontal section profile on the most protrusion of breast, which is processed as a convex hull. |
| Chest circumference (cm) | The length of the horizontal section profile on the most protrusion of breast, which is processed as a convex hull. |
| Waist width (cm) | The width of the horizontal section profile on the belly button, which is processed as a convex hull. |
| Waist circumference (cm) | The length of the horizontal section profile on the belly button, which is processed as a convex hull. |
| **Hip** |  |
| Hip width (cm) | The width of the horizontal section profile on the most protrusion of hip, which is processed as a convex hull. |
| Hip circumference (cm) | The length of the horizontal section profile on the most protrusion of hip, which is processed as a convex hull. |
| **Upper limbs** |  |
| Left arm length (cm) | The length from the end of the left clavicle, where the left arm and the shoulder meet, to the left wrist. |
| Right arm length (cm) | The length from the end of the right clavicle, where the right arm and the shoulder meet, to the right wrist. |
| Left upper arm circumference (cm) | The length of the section profile on middle point of left upper arm, which is perpendicular to the axis of humerus and processed as a convex hull. |
| Right upper arm circumference (cm) | The length of the section profile on middle point of right upper arm, which is perpendicular to the axis of humerus and processed as a convex hull. |
| Left forearm circumference (cm) | The length of the section profile on middle point of left forearm, which is perpendicular to the axis of left forearm and processed as a convex hull. |
| Right forearm circumference (cm) | The length of the section profile on middle point of right forearm, which is perpendicular to the axis of right forearm and processed as a convex hull. |
| **Lower limbs** |  |
| Left leg length (cm) | The length from the crotch interaction to the heel of left leg. |
| Right leg length (cm) | The length from the crotch interaction to the heel of right leg. |
| Left thigh circumference (cm) | The length of the horizontal section profile on 2/3 height. of left thigh, which is processed as a convex hull. |
| Right thigh circumference (cm) | The length of the horizontal section profile on 2/3 height of right thigh, which is processed as a convex hull. |
| Left knee circumference (cm) | The length of the horizontal section profile on the center of left kneecap, which is processed as a convex hull. |
| Right knee circumference (cm) | The length of the horizontal section profile on the center of right kneecap, which is processed as a convex hull. |
| Left calf circumference (cm) | The length of the horizontal section profile on 2/3 height of left leg, which is processed as a convex hull. |
| Right calf circumference (cm) | The length of the horizontal section profile on 2/3 height of right leg, which is processed as a convex hull. |
| * Adapted from Chuang et al.(2006) and Lin et al.(2002) (17, 19). | |
